# Supplementary material for: Internal and external validity of the brief version of the Multidimensional Personality Questionnaire: Exploratory structural equation modelling
Source: PLoS One. 2020 Apr 6;15(4):e0229470. doi: 10.1371/journal.pone.0229470 (PMC7135061; doi:10.1371/journal.pone.0229470)
Supplement: Attachment — Submitted filename: response to Review Comments to the Author.docx [file pone.0229470.s003.docx]

**Response to Review Comments to the Author**

**Reviewer #1:**

Even though the results presented in the paper are interesting it is not stressed enough which may be their practical implications. The Authors suggested using a “reduced version” of the 11 primary lower-order trait scales (p 21): Would be reliable these scales? Which may be the results of using the reduced scales in terms of construct validity? Which may be the meaning of a non-defined structure at the item-level? How could be overcome these problems of the scale? Is it a problem of the scale or a matter of the underlying theory?

Response. Discussed in page 21 , para 1.

In addition, I would suggest to the Authors to consider shortening the paper by simplifying some paragraphs (e.g., first paragraph p 21, p 16). To this purpose, some tables would be useful.

Response. The section referred to has been shortened, with a new table (Table 3) to identify the relevant items (p. 21 para 2)

Please, control the t-test (p 12).

Response. No t-test findings was reported in the paper.

**Reviewer #2:**

Nevertheless, the preference to this theory, or its advantages compared to other main personality theories and assessment methods are not entirely and clearly explained. In order to strengthen the manuscript, I would suggest the authors to add a paragraph addressing this issue to highlight the utility of the MPQ in the introduction.

Respone. This is provided in the revised paper (p. 7, para 2).

The main weakness of this work involves the relatively small although normative sample recruited by the authors. I would suggest that the authors highlight more clearly this fact as a limitation in their limitation section.

Response. This is cover in p. 22, para 2 (fifth limitation).

Considering the implications, although briefly highlighted, I would suggest that the authors include a specific part about how the present findings discussing how they could inform Tellegen's theory in regards to specific potential conceptual modifications-reconsiderations.

Response. Covered in p. 21, para 1.
